# A Novel Renoprotective Strategy: Upregulation of PD-L1 Mitigates Cisplatin-Induced Acute Kidney Injury

**DOI:** 10.3390/ijms222413304

**Published:** 2021-12-10

**Authors:** Jun Liu, David C. Yang, Jun Zhang, Ssu-Wei Hsu, Robert H. Weiss, Ching-Hsien Chen

**Affiliations:** 1 Department of Internal Medicine, Division of Nephrology, University of California Davis, Davis, CA 95616, USA; jliuucd@gmail.com (J.L.); dvcyang@ucdavis.edu (D.C.Y.); jmzhang@ucdavis.edu (J.Z.); suwhsu@ucdavis.edu (S.-W.H.); rhweiss@ucdavis.edu (R.H.W.); 2 Department of Internal Medicine, Division of Pulmonary and Critical Care Medicine, University of California Davis, Davis, CA 95616, USA; 3 Comprehensive Cancer Center, University of California Davis, Davis, CA 95616, USA

**Keywords:** immune checkpoint, AKI, T cells, inflammation, renal tubules

## Abstract

The innate and adaptive immunities have been documented to participate in the pathogenesis of nephrotoxic acute kidney injury (AKI); however, the mechanisms controlling these processes have yet to be established. In our cisplatin-induced AKI mouse model, we show pathological damage to the kidneys, with the classical markers elevated, consistent with the response to cisplatin treatment. Through assessments of the components of the immune system, both locally and globally, we demonstrate that the immune microenvironment of injured kidneys was associated with an increased infiltration of CD4+ T cells and macrophages concomitant with decreased Treg cell populations. Our cell-based assays and animal studies further show that cisplatin exposure downregulated the protein levels of programmed death-ligand 1 (PD-L1), an immune checkpoint protein, in primary renal proximal tubular epithelial cells, and that these inhibitions were dose-dependent. After orthotopic delivery of PD-L1 gene into the kidneys, cisplatin-exposed mice displayed lower levels of both serum urea nitrogen and creatinine upon PD-L1 expression. Our data suggest a renoprotective effect of the immune checkpoint protein, and thereby provide a novel therapeutic strategy for cisplatin-induced AKI.

## 1. Introduction

Acute kidney injury (AKI) is a clinical problem, defined by a rapid decline in renal function occurring within a short-term time span of a few hours to a few days. It is marked by tubular epithelial damage, with resultant damage and the loss of kidney function, causing an accumulation of waste products, and a loss of electrolytes and the acid-base balance [1]. AKI is a significant clinical problem, with 4 million hospitalization cases in 2014, and this number continues to grow year every year. This troubling increase indicates a growing health concern and underscores the importance of understanding the pathogenic mechanisms of this problem [2]. Of the many wide-ranging and often multifactorial causes of AKI, drug nephrotoxicity represents a significant portion of cases in the clinical setting, accounting for between 8–60% of AKI cases, depending on the cohort [3]. A large multicenter study indicated that up to 19% of AKI cases could be attributed to drug toxicities [4], further supporting this observation. This could primarily be attributed to the function of the kidney as a filter; thus, it is a major target site that is exposed to the toxic effects of therapeutic drugs and their metabolites as the kidney performs its normal function in filtering the circulating blood.

Cisplatin (cis-diammine-dichloro-platinum II), and other platinum chemotherapy drugs, are among the most widely used chemotherapeutic drugs for a diverse range of solid and liquid tumors [5]. Although effective, the usage of cisplatin and other platinum-based drugs has a broad range of significant side effects, including nephrotoxicity. Up to one-third of patients experience some degree of nephrotoxicity after just one dose of cisplatin [6]. Thus, the evaluation of the mechanisms of damage induced by cisplatin exposure, and the development of strategies to mitigate this damage are warranted to improve patient outcomes and to potentially expand the utility of cisplatin through a reduction in the nephrotoxic side effects.

One potential approach is to target the inflammatory processes evoked in response to chemotherapeutic treatment. Most AKI has been demonstrated over recent years to be a systemic disease, with inflammation playing a critical part in the disease process [7]. After the initial injury of the tubular epithelium, inflammatory cells are recruited to the site of the injury. Virtually all immune cells are involved in the process, with both adaptive and innate immunities shown to contribute to the pathogenesis of AKI [7,8,9]. Given the importance of the immune system in regulating the response to kidney injury in response to toxic agents and the prevalence of cisplatin in the standard of care in cancer, it is of distinct interest to evaluate how the immune system modulates the damage associated with cisplatin-induced AKI.

Essentially all of the components of the immune system have been implicated as playing roles of variable importances and effects in nephrotoxic AKI, but the mechanisms controlling these processes are still not completely understood. In this study, we show the immune microenvironment of cisplatin-injured kidneys and demonstrate the therapeutic potential of genetically engineered kidneys with PD-L1 expression in mitigating cisplatin-mediated kidney damage.

## 2. Results

### 2.1. Cisplatin Induces Acute Kidney Injury

**Figure 1:**
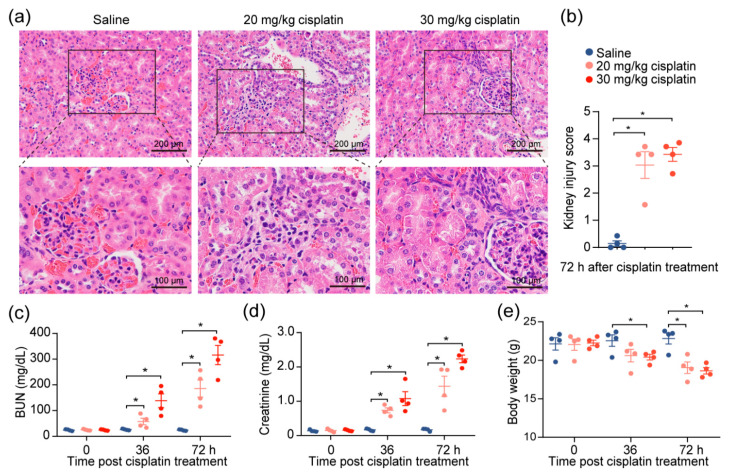
Cisplatin induces acute kidney injury. Mice were intraperitoneally injected with one dose of cisplatin, at 20 mg/kg or 30 mg/kg, for 72 h. Body weight, levels of BUN, and creatinine in serum were determined at 36 and 72 h. At 72 h, spleens and kidneys from mice were collected for pathology analysis and immunophenotyping, respectively. (**a**) Representative images, and (**b**) tubular injury quantification of H&E staining in kidney specimens from mice treated with 30 mg/kg cisplatin for 72 h (*n* = 4). (**c**–**e**): levels of serum BUN (**c**), creatinine (**d**), and body weight (**e**) (*n* = 4. * p < 0.05).

Given the observations of kidney injury in response to cisplatin, we investigated the effects of cisplatin on renal function by modifying a previously established cisplatin-induced AKI mouse model [10]. After the intraperitoneal administration of 20 or 30 mg/kg of cisplatin, we confirmed severe renal damage in mice injected with a single dose of cisplatin, as indicated by the distortion of the tubules and tubular cell necrosis (Figure 1a,b). We also noted an expected increase in the level of serum blood urea nitrogen (BUN) and creatinine, which increased with escalating doses (Figure 1c,d), reflecting the renal damage caused by cisplatin. To account for the possibility that diet and muscle mass content could influence the serum creatinine and BUN [11], we evaluated the change in the body weights of the mice throughout the duration of the experiment. We found that, despite weight reduction in cisplatin-treated mice, there was no correlation between creatinine levels and weight loss (Figure 1e). The data demonstrate that chemotherapeutic drug cisplatin decreases kidney function.

### 2.2. AKI Mice Exhibit an Imbalanced Immune Response

**Figure 2:**
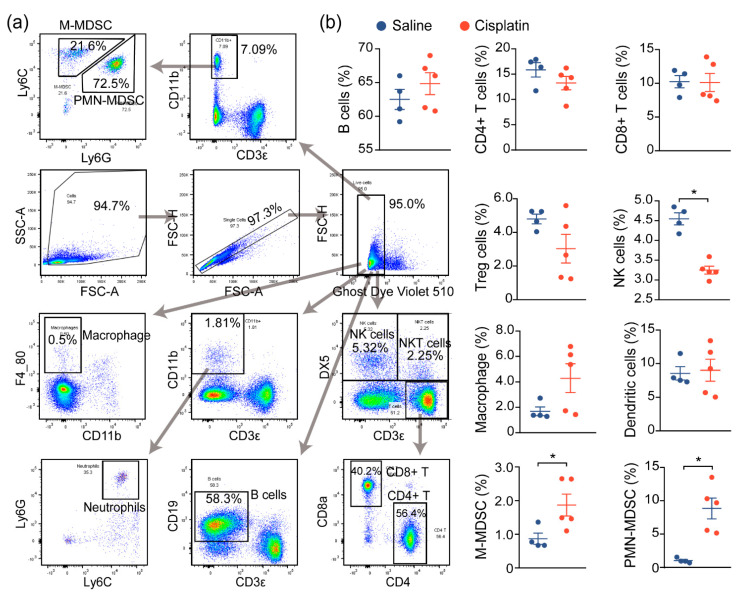
AKI mice exhibit an imbalanced immune response. (**a**) Gating strategy for immune cell profiling of mice in Figure 1. Single cell suspension of immune cells from spleens of mice were prepared and labeled with the indicated antibodies and dyes. Data were acquired by flow cytometry and analyzed using Flowjo. (**b**) Analysis of splenic immune cells in mice administered with saline (*n* = 4), or 30 mg/kg cisplatin (*n* = 5, * p < 0.05).

Prior reports have demonstrated that immune cell infiltration and a proinflammatory immune response contribute to the damage and subsequent renal dysfunction during AKI. In agreement with prior reports, we noted increased immune cell infiltration in cisplatin-exposed kidneys, compared to nonexposed kidneys, and we next assessed the changes in the immune profile and functionality of these cells, both locally and globally. We first evaluated the global inflammatory status by analyzing the immune cells in the spleen, a critical immune organ involved in local and systemic inflammation [12]. Flow cytometry analysis demonstrated a significant change in the populations of monocytic and polymorphonuclear myeloid-derived suppressor cells (M-MDSC and PMN-MDSC), as well as natural killer (NK) cells, although no significant changes on the B cells, T cells (CD4+, CD8+, Treg), macrophage, or DCs were observed between saline- and cisplatin-treated mice. This suggests that the immune response may be dysregulated in response to cisplatin exposure (Figure 2a,b).

### 2.3. Immune Characterization and Profiles of AKI

**Figure 3:**
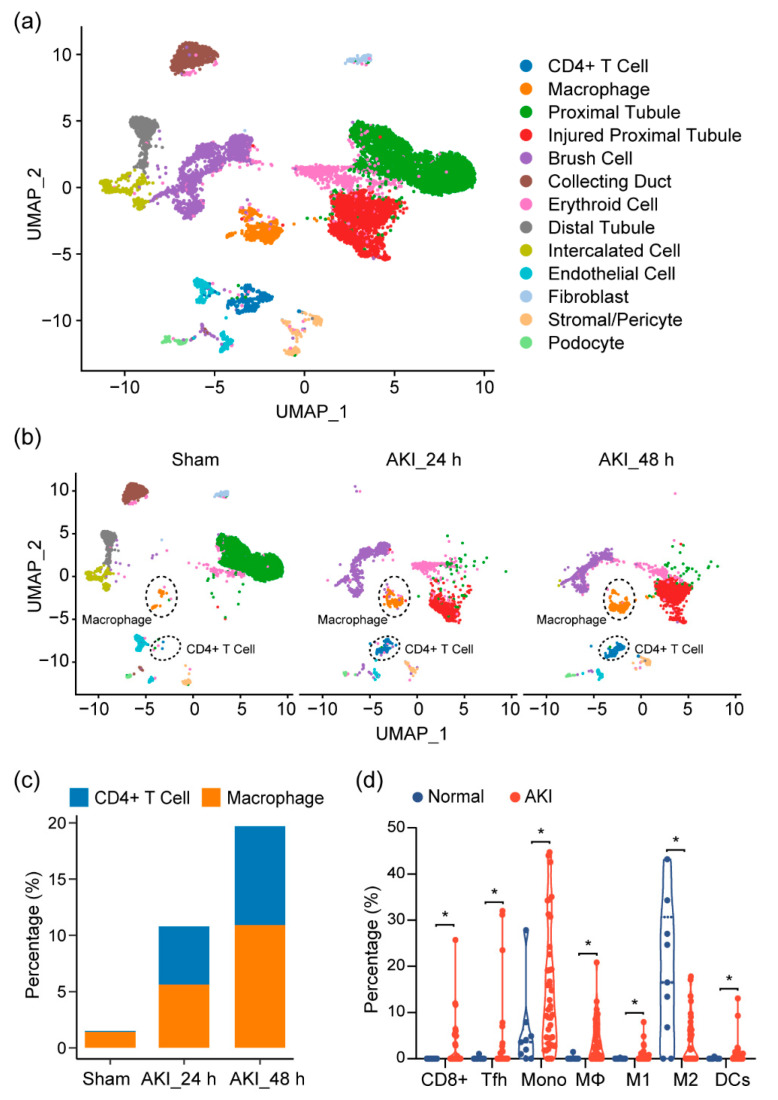
Immune characterization and profiles of AKI. (**a**) UMAP clustering of cell types in kidneys, from normal and AKI mice, by analysis of single-cell RNA-sequencing data (GSE139506). (**b**) Cell-type distribution in kidneys, from normal and AKI mice, 24 h and 48 h after induction of AKI. (**c**) Quantification of CD4+ T cells and macrophage cells, as percentage of total cell populations, in kidneys from normal and AKI mice. (**d**) Estimated proportion of the immune subpopulations in human kidney biopsy samples (GSE139061) using CIBERSORT analysis. Data are presented as mean ± SEM. AKI (*n* = 39) and normal (*n* = 9), * p < 0.05.

Given our observations, as well as previous reports showing that the immune profile is altered in cisplatin-induced AKI, we sought to look at the shifts in the cell population on the single-cell level in order to provide a more comprehensive assessment of the immune response in AKI and to further elucidate the cell types at play in this process. At the time of writing, single-cell data for cisplatin-induced AKI did not exist. Thus, we turned to single-cell RNA sequencing (scRNA-seq) data on mouse kidneys for ischemia/reperfusion (IR)-induced AKI, a model that resembles drug-induced AKI and shares many common aspects in the immune response. After quality control, 11,682 cells passed filtration and were subjected to clustering and dimensionality reduction with Uniform Manifold Approximation and Projection (UMAP). Clusters were further annotated by directly comparing their transcriptional profile with known cell-type-specific markers. We identified 13 cell populations, including proximal tubular cells, injured proximal tubular cells, etc. (Figure 3a). In the immune compartment, we observed elevations of the CD4+ T cells and macrophages during AKI progression (Figure 3b,c).

Next, to see if this could be observed in human tissue, we analyzed RNA-seq data of kidney biopsy tissue in dataset GSE139061. Through cell-type identification, by estimating the relative subsets of RNA transcripts through CIBERSORT, we noted that T cells, as well as macrophage cells, were the predominantly elevated immune cell populations in AKI, confirming our findings in mouse tissue (Figure 3d). In particular, we noted proinflammatory cell types (CD8+ cells, Tfh, M1, and DC cells). These elevations were also mirrored by a decrease in the M2 macrophage population, suggesting a more inflammatory environment with a decrease in reparative/anti-inflammatory cell types.

### 2.4. PD-L1 Dysregulation in Renal Epithelium upon Kidney Injury

**Figure 4:**
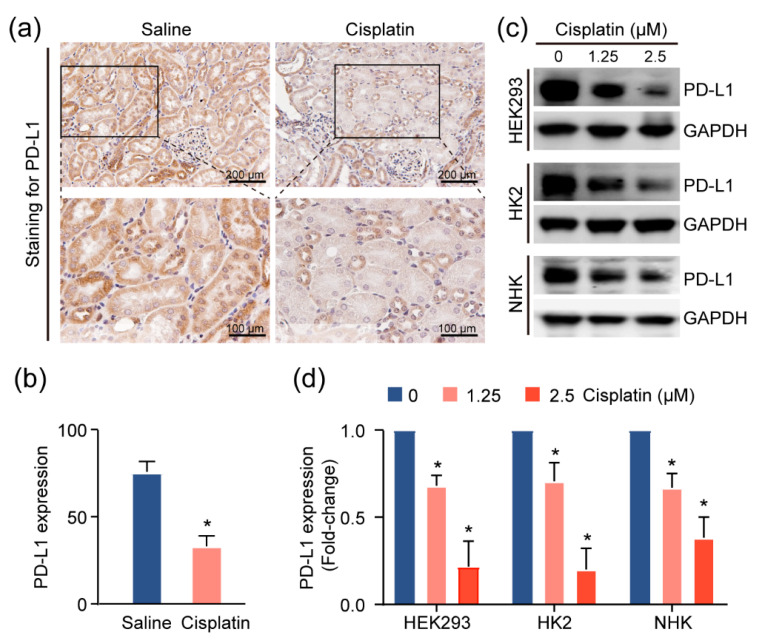
PD-L1 dysregulation in renal epithelium upon kidney injury. (**a**) Representative images for PD-L1 expression levels and quantification, (**b**) of PD-L1, assessed by immunohistochemical (IHC) staining in cisplatin-exposed kidneys (*n* = 4) using anti-PD-L1 monoclonal antibody. Positive staining is quantified (mean ± SD, * p < 0.05 versus saline group). (**c**) Western blot analysis of kidney cell lines treated with escalating doses of cisplatin and quantification, (**d**) for PD-L1 levels in cells upon treatment with cisplatin for 24 h. Normalized expression levels of PD-L1 from three separate experiments are shown (* p < 0.05).

Given the increase in the T-cell population in injured kidneys, we suspected that these cells contribute to the inflammatory microenvironment in the kidney. As the activities of these T cells are associated with the expression of the immune checkpoint molecules, particularly programmed death ligand 1 (PD-L1) [13], we utilized IHC assays to assess the levels of this immune checkpoint protein in the CAKI model mouse tissue. We observed more intense PD-L1 staining in normal tubular epithelial cells, as compared to cisplatin-exposed renal mouse epithelium (Figure 4a,b). Consistently, treatment with cisplatin reduced PD-L1 expression in HEK293 cells, and two primary human kidney epithelial cell lines, HK2 and NHK (Figure 4c,d), suggesting the possibility that the inflamed kidney microenvironment is attributable to cisplatin-induced PD-L1 downregulation in the renal epithelium.

### 2.5. Targeting PD-L1 Protects against AKI

**Figure 5:**
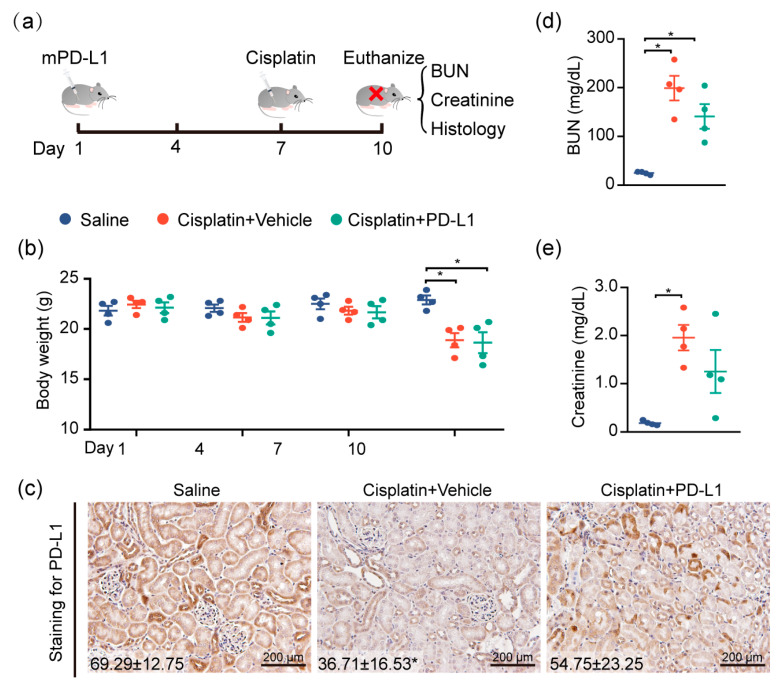
Targeting PD-L1 protects against AKI. PD-L1-containing lentiviruses were delivered into the kidneys of mice through subcapsular injection. Seven days after injection, mice were intraperitoneally treated with 30 mg/kg cisplatin for 72 h, and body weights and blood sera were collected at the end of the study, at 10 days. (**a**) Study timeline. (**b**) Body weights of mice throughout the duration of the experiment. (**c**) Representative images of IHC staining using anti-PD-L1 antibody (*n* = 4; mean ± SD). (**d**) The levels of serum BUN and (**e**) creatinine were determined (*n* = 4; * p < 0.05).

**Figure 6:**
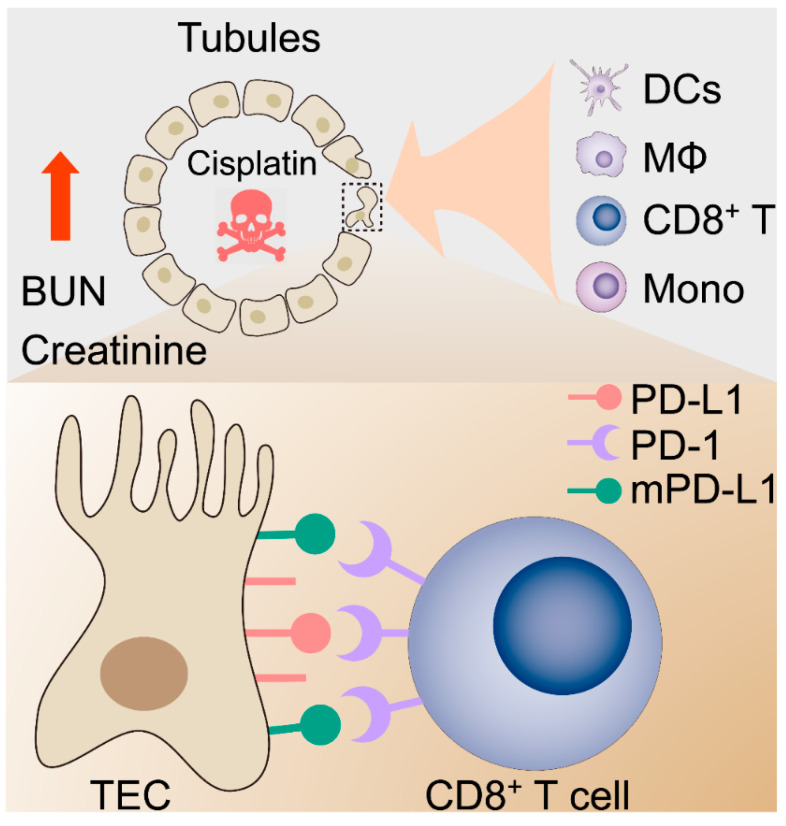
Proposed hypothetical model for the contribution of the kidney immune microenvironment and the PD-1/PD-L1 in nephrotoxic AKI.

To test the renoprotective effect of PD-L1 expression, the mouse kidney was genetically engineered by utilizing the lentivirus-mediated overexpression of mouse PD-L1 (mPD-L1) prior to cisplatin exposure. Figure 5a illustrates the protocol for cisplatin administered in this study. IHC staining confirmed that kidneys receiving mPD-L1 lentivirus participles rescued the expression level of PD-L1 in tubular epithelial cells after 72 h of cisplatin exposure (Figure 5c), whereas an approximately 2-fold decrease in the PD-L1 level still occurred in the cisplatin-alone group. Surprisingly, the overexpression of mouse PD-L1 decreased the cisplatin-induced serum BUN and creatinine (Figure 5d,e), while no significant difference was noted in the weight loss between the two cisplatin-treated groups (Figure 5b). Altogether, our results show, for the first time, a critical role for PD-L1 expression in the renal epithelium in the regulation of the kidney immune microenvironment and the improvement of kidney function (Figure 6).

## 3. Discussion

### 3.1. Activated Immune System during AKI

It is widely understood that the immune system plays a critical role in modulating the processes that occur during nephrotoxic AKI, with both the adaptive and innate immune cells mediating the process. To assess the functionality of the components of the immune system during cisplatin-induced AKI, we investigated the immune profiles, both locally and globally, during cisplatin-induced kidney injury. We first assessed the global inflammatory status by analyzing the immune cells in the spleen. The spleen, a critical immune organ, plays roles in local and systemic inflammation, and offers a window into the status of the immune system [12].

In our analysis of the immune cells in the spleen, we observed an activated immune system, with the beginnings of the repair and resolution processes mediated through immune cells. We also noticed an increase in MDSCs, which have an immunosuppressive effect [14]. Although we did not assess the changes in MDSCs over the time course of cisplatin-induced AKI, the elevations of MDSCs are likely induced responses to the elevated inflammatory mediators present because of injury. Furthermore, increases in the total macrophages were noted in both the mouse and human AKI datasets (Figure 3b–d). Macrophages have been shown to be contributors to the inflammatory process in AKI [7,15]. In particular, M2-polarized macrophages have been demonstrated to reduce damage in AKI and mediate the repair process. However, the macrophages observed in this current work may also be proinflammatory M1 macrophages, as suggested by the elevation of the M1 population in human AKI (Figure 3d).

In the spleen, a significant decrease in the NK cells occurred in response to the exposure to cisplatin. NK cells typically have high cell numbers in the spleen and have been demonstrated to be able to migrate to other tissues; therefore, this decrease may indicate that the cells have migrated to the site of inflammation [16,17]. Taken as a whole, we observed the mobilization of both innate and adaptive immune cells within our mouse model, pointing to activated immune processes occurring, with both proinflammatory and reparative processes at play, during cisplatin-induced AKI.

### 3.2. Immune Changes in the Kidney

Given our findings in the splenic immune cells, we looked further into the immune profile at the site of injury caused by cisplatin: the kidney. We performed an analysis of the mouse single-cell mRNA sequencing data, as well as a bioinformatic analysis (CIBERSORT) of the RNAseq data of kidney tissue from human AKI (GSE139061). Within the mouse AKI single-cell data (Figure 3a), we observed an expansion of macrophages and CD4+ T cells, concomitant with injured tubule cells, indicating an expansion of these immune cells during injury.

Consistent with our earlier findings within the splenic immune cells, and with findings from other groups, we noted an increase in the macrophage population. Monocytes/macrophages seem to be significant players in AKI pathogenesis. Upon AKI, monocyte/macrophage trafficking to the site and the proinflammatory milieu near the site promoting M1 polarization, has been previously demonstrated to function in mediating AKI. The proinflammatory environment polarizes these macrophages toward a proinflammatory (M1) phenotype, propagating inflammation and furthering damage in the kidney [7,15]. The attenuation of these macrophages in the early stages (inflammatory phase) of AKI has been shown to be protective [7,15]. This sequence of events suggests that the expanded macrophage population we noted in the mouse kidney immune cell data is likely to consist of proinflammatory M1 macrophages. Supporting this is the consensus that M1 macrophage populations are elevated during the first three days of AKI, with M2 macrophages showing up later in the process after three days [15]. Further support of the proinflammatory polarization of macrophages is seen in the data in human AKI samples. The M1 macrophage population is expanded in AKI kidney tissue, with a concurrent decrease in M2 macrophages (Figure 3d).

Looking further into the human AKI RNA-seq data, we also noted the expansion of dendritic cells. These cells have been shown to play a protective role in nephrotoxic AKI, seemingly suppressing inflammation [7,8,18], and may be a response to the damage induced by cisplatin. However, it is worth considering that DC cells have important roles as antigen-presenting cells and are found at sites of injury and inflammation. DC-cell elevation in the kidney during AKI, along with an elevation in macrophages, could potentially drive increased antigen presentation and support adaptive immune responses to AKI. Indeed, DC cells have been shown to be detrimental in some models of AKI [19].

### 3.3. T-Cell Changes in AKI

Beyond macrophages and DC cells, changes in the T-cell compartment were remarkably noted during AKI. Mouse AKI scRNA-seq data confirmed an increase in the CD4+ cell population in the kidney (Figure 3b). Prior studies have pointed to the adaptive immune system, with T cells, in particular, demonstrating roles in controlling the AKI process. Within the adaptive immune compartment, CD4+ and CD8+ T cells both have been studied extensively for their role in AKI. Within the CD4+ compartment, there are broadly Th1, Th2, Th17, Treg, and Tfh cells. Th1 and Th17 cells have been shown to play a more outsized role in mediating damage, given their role in inflammation. Blockage of the Th1 response protects against AKI and Th17 cells, which have been shown to be the most abundant infiltrating lymphocytes during AKI [8,20,21].

Th2 cells seem to play a less significant role but have been shown to participate in the resolution of AKI [20]. These cells may be similar to M2 macrophages in the sense that they are likely part of the reparative processes that occur after injury. With regard to the Treg cells, they have been shown to be protective in AKI through the modulation of the inflammatory response [7,8,9,21], but we did not notice a significant elevation of this population within human AKI. We did observe a significant increase in the Tfh cells in human AKI (Figure 3d). These cells play a role in humoral immunity and may play a role in AKI, albeit we did not note significant changes to the B cells within our data. Given that most reports indicate that most of the cells infiltrating the kidney during AKI are proinflammatory, most of the cells we noted in the single-cell data (Figure 3b,c) are likely proinflammatory Th1 and Th17 cells. We also found an increase in CD8+ T cells in human AKI (Figure 3d). These cells have been demonstrated to contribute to cisplatin-induced AKI [22,23], although to a lesser extent than CD4+ T cells. Given the marked increase, these cells may be playing an underappreciated role in cisplatin-induced AKI.

### 3.4. The PD-1/PD-L1 Axis in Cisplatin-Induced AKI

With the changes observed in CD4+ and CD8+ T cells, we turned our investigation towards the PD-1/PD-L1 axis, as this immune checkpoint plays a critical role in modulating immune cell activities. In particular, this notion is supported by clinical observations that report that AKI occurred in roughly 2–3% of the patients treated with immune checkpoint inhibitors (ICIs), such as PD-1, PD-L1, and CTLA-4 inhibitors, with a meta-analysis of data from other clinical trials supporting these findings [24,25]. This is potentially mediated through the breakage of self-tolerance, as self-reactive T cells are no longer restricted by checkpoint inhibition. The mechanisms of this process are still poorly understood; thus, we investigated if this axis could be mediating a role in cisplatin-induced nephrotoxic AKI.

Previous studies have pointed to the vital role of the PD-1/PD-L1 axis in modulating the immunogenic activities of the T cells [26,27], as well as other immune cell types. Foremost, this axis regulates CD8+ T cell activity and controls the cytotoxic-cell-killing ability of these cells. The axis also plays a role in limiting CD4+ cell responses to immune stimuli [28]. Beyond this, PD-1/PD-L1 has also been shown to regulate macrophage activity with the suppression of PD-L1 in macrophages, leading to a decreased M2 phenotype and an increased M1 phenotype. Additionally, it has also been shown that the activation of PD-1 on macrophages upregulates M2 polarization, with a subsequent decrease in the M1 phenotype. Thus, the blockade of PD-1/PD-L1 may not only allow unrestrained CD4+/CD8+ T-cell activity but may also promote proinflammatory phenotypes in macrophages, potentially dysregulating the inflammatory response.

Bearing this in mind, we assessed the role of this axis in cisplatin-induced AKI. We noted that, in cisplatin-treated kidneys, PD-L1 was significantly reduced, and this was further supported by our findings in vitro (Figure 4c,d). The ectopic expression of PD-L1 in the kidney rescued the PD-L1 expression and reduced the markers of kidney damage. Although ectopic PD-L1 expression was not able to completely protect against kidney injury, we do see a significant decrease in injury. This is likely due to the reduced CD4+/CD8+ T-cell activity, as well as to the reduced proinflammatory macrophage activity. However, further analyses of the immune cell types at play, and their phenotypes after PD-L1 ectopic expression, are warranted in order to further confirm these observations.

### 3.5. Concluding Remarks

In all, we have demonstrated an important role of PD-L1 in modulating inflammation in response to nephrotoxic AKI induced by cisplatin. Given this role in controlling inflammation, and the observation of AKI in the clinical setting due to ICI therapies, PD-1/PD-L1 targeting therapies, in conjunction with nephrotoxic drugs (such as the widely used cisplatin), need to be critically evaluated to avoid unintended kidney injury. The finding that PD-L1 levels are decreased in cisplatin-induced nephrotoxicity is interesting, as previous reports have indicated that cisplatin can increase the PD-L1 levels in cancers [29], with this phenomenon being an active area of investigation [30]. This is a complex issue; there is a need to balance the immune activation against tumors while controlling the immunological activity against the self. Approaches, such as engineered PD-L1-expressing DC cells, or PD-L1 carrying adenovirus delivery into the kidney to tamp down immune activation in the region, may also be approaches of consideration However, they require precise delivery to the kidney, as the elevation of PD-L1, and the subsequent immunosuppression, may hinder concurrent cancer therapy [31]. A thorough consideration of the costs and benefits to patients, a careful balancing of immune activation by ICIs with cisplatin therapy, as well as an exploration of alternative strategies (e.g., targeted radiation, precise surgical excision, where possible) could help lessen potential injury and inflammation in the kidney.

## 4. Materials and Methods

### 4.1. Mouse Model of Cisplatin-Induced AKI and PD-L1 Orthotopic Expression

Eight-week-old C57BL/6J mice, purchased from the Jackson Laboratory, were housed and fed food and water ad libitum. Prior to treatment with pharmaceutical-grade cisplatin (Millipore Sigma, Burlington, MA, USA), mice were randomly divided into one control group (saline) and two cisplatin-treated groups. Mice in the latter two groups were intraperitoneally injected with one dose of cisplatin, at 20 mg/kg or 30 mg/kg, for 72 h. Body weights and levels of BUN and creatinine in sera were determined at 36 and 72 h. For PD-L1 expression in vivo, mice were orthotopically injected, either with Lenti ORF control particles or PD-L1 (CD274) mouse-tagged ORF clone lentiviral particles (OriGene Technologies, Inc., Rockville, MD, USA), into the kidney, as we described previously [32]. After the animals had recovered from the procedure, these mice were treated with either saline or 30 mg/kg of cisplatin. At 72 h after saline or cisplatin administration, the mice were sacrificed, and the tissues and blood were collected. Kidney and spleen tissues were collected for histological and flow cytometry analysis, and blood sera were collected for assaying the creatinine and BUN levels. All mouse experiments were approved by the Institutional Animal Care and Use Committee (IACUC) of UC Davis.

### 4.2. Hematoxylin and Eosin (H&E) Staining Measurement

Paraffin-embedded kidney tissue sections were cut and stained with H&E, according to the standard staining protocols for histological analysis. Tubular injury degeneration was defined as including vacuolization, luminal cell casts, and acellular/atrophic changes [33]. Tubular injury scoring was as follows: 0 = none detected; 1 = 1–10% tubules involved; 2 = 10–25% tubules involved; 3 = 25–50% tubules involved; and 4 = >50% tubules involved. These results were reviewed and scored by an experienced kidney pathologist who was blinded to the identities of the samples.

### 4.3. Immunohistochemistry

For immunohistochemical staining, detailed experimental procedures were modified from the paraffin immunohistochemistry protocol supplied by the manufacturer (Cell Signaling Technology, Inc., Danvers, MA, USA), as described previously [34]. The slides were deparaffinized in a xylene substitute and rehydrated in graded alcohol and water, followed by an antigen-retrieval step (10 nM sodium citrate with pH 6.0, at a sub-boiling temperature). Endogenous peroxidase activity was blocked by 3% hydrogen peroxide, followed by blocking serum and incubation with anti-PD-L1 antibody (Cell Signaling Technology, Inc., Danvers, MA, USA) overnight at 4 °C. Detection of immunostaining was conducted using the VECTASTAIN^®^ ABC system, according to the manufacturer’s instructions (Vector Laboratories, Burlingame, CA, USA).

### 4.4. Cell Culture

HEK293 and HK2 cells were purchased from the American Type Culture Collection (ATCC) (Manassas, VA, USA). Normal human primary kidney tubular epithelial cells (NHK cells, also known as human renal proximal tubule epithelial cells (RPTEC)) were purchased from Lonza (Allendale, NJ, USA) [35,36]. All cells were cultured in Dulbecco’s modified Eagle’s medium (DMEM), supplemented with 10% fetal bovine serum and 1% penicillin-streptomycin, at 37 °C, in a humidified atmosphere of 5% CO_2_.

### 4.5. Western Blot Analysis

Detailed procedures for the Western blot analyses and the preparations of the whole-cell lysates have been described previously [33,37,38]. In brief, whole-cell lysates were prepared by lysing cells in a lysis buffer (50 mM Tris-HCl (pH 7.4), 1% NP-40, 150 mM NaCl, 1 mM EDTA, 20 μg/mL leupeptin, 1 mM PMSF, and 20 μg/mL aprotinin), and proteins were denatured with heat and SDS. Prepared lysates were then subjected to separation by SDS-PAGE. Immunoblotting was conducted with appropriate antibodies, followed by chemiluminescent detection.

### 4.6. Flow Cytometry Assays

Spleens were removed from euthanized animals, and single-cell suspensions of leukocytes were prepared by disaggregation of the tissue through a 100-μm nylon mesh, followed by dissociation, as we reported previously [39]. Cells were washed once with complete DMEM media, supplemented with 10% heat-inactivated FBS (hiFBS), then incubated with RBC lysis buffer for 1 min to remove red blood cells, then washed again in complete DMEM media. Cell isolates were prepared, as above, then washed and resuspended in staining medium (1× PBS, 0.5% BSA, 0.02% sodium azide). Ghost Dye Violet 510 was used for live-/dead-cell staining. Leukocytes were labeled with a combination of the following antibodies: PE-Cy5 CD3ε, Brilliant Violet 570 CD4, PE/Cy7 CD8a, APC-Fire750 CD11b, Brilliant Violet 785 CD19, PE/Dazzle 594 DX5, Alexa Fluor 700 F4/80, Brilliant Violet 650 Ly-6C, and Brilliant Violet 711 Ly-6G (BioLegend, San Diego, CA, USA). Gating strategy is as shown in Figure 2a. Data were acquired and analyzed using LSR II flow cytometry and Flowjo software (BD Biosciences, Franklin Lakers, NJ, USA).

### 4.7. Single-Cell mRNA Sequencing (scRNA-seq) Data Analysis

Transcriptomes of single cells from normal and injured mouse kidneys from the GEO dataset (GSE139506) were analyzed [40]. Briefly, the raw counts of the mRNA sequencing were subjected to quality control (QC) and normalization using the Seurat package (version 3.1.0) in R (version 4.0.1). A total of 11,682 cells passed filtration by discarding cells expressing ≥40% of mitochondria gene expression (percent.mt). Principal component analysis (PCA) was performed on the top 2000 most variable genes, and the first 20 PCAs were used for downstream analysis. Dimensional reduction was performed using Uniform Manifold Approximation and Projection (UMAP). Cell types were identified using cluster-specific genes, by mapping Mouse Cell Marker (http://biocc.hrbmu.edu.cn/CellMarker/index.jsp; (accessed on 12 September 2021).

### 4.8. Statistical Analysis

Data are presented as the mean ± SD for at least three independent experiments. The statistical analyses for quantitative in vitro and in vivo data were performed using the student’s *t*-test. All analyses were performed using SPSS software (v10.0; SPSS, Inc., Chicago, IL, USA). All statistical tests were two-sided, and *p* values < 0.05 were considered statistically significant.

## Data Availability

The data presented in this study are available upon request from the corresponding author.
